# Sacral Erector Spinae Plane Block Provides Surgical Anesthesia in Ambulatory Anorectal Surgery: Two Case Reports

**DOI:** 10.7759/cureus.12598

**Published:** 2021-01-09

**Authors:** Cengiz Kaya, Burhan Dost, Serkan Tulgar

**Affiliations:** 1 Anesthesiology and Reanimation, Ondokuz Mayıs University, Samsun, TUR; 2 Anesthesiology, Maltepe University Faculty of Medicine, Istanbul, TUR

**Keywords:** nerve block, ultrasonography, sacrum, anal fistula

## Abstract

Erector spinae plane block (ESPB) is a new and popular interfacial fascial plane block which has been used in many different surgeries. There are a few cases in which ultrasound-guided sacral ESPB was used for postoperative analgesia. This article presents the successful use of bi-level, bilateral sacral ESPB for main anesthetic method in anorectal surgery. Anesthetic level required for surgery was accomplished in 30 minutes, and none of the patients experienced pain throughout the surgery. The patients were discharged at the postoperative fourth hour without any complications. The patients, who were contacted later, indicated no need for any analgesic for 24 h postoperatively. To the best of our knowledge, this is the first case report in the literature where sacral ESPB is used as the sole anesthetic technique. The sacral ESPB can be considered in anorectal surgery as an alternative technique for spinal or general anesthesia.

## Introduction

Although the erector spinae plane block (ESPB) is a relatively new interfacial fascial plane block, it has become widely used in a short time for postoperative analgesia in different surgeries. The lumbar ESPB has been described for gender reassignment procedures, vaginismus and abdominal surgeries [1,2]. Also, recently, lumbar ESPB has been used successfully as the main anesthetic method for hip surgery [3]. An examination of the anatomy of the erector spinae muscle group reveals that it extends from the cervical to the sacral region; therefore, at least theoretically, ESPB can be performed at different levels along this entire vertebral column [4]. However, apart from a few case reports of using ultrasound (US)-guided sacral ESPB for postoperative analgesia, as first described by Tulgar et al., its use for anesthesia alone has not been reported in the available literature [5,6].

This article presents the successful use of bi-level, bilateral sacral ESPB alone for anesthesia in anorectal surgery. To the best of our knowledge, this is the first case report in the literature where sacral ESPB is used as the sole anesthetic technique. Written informed consent was obtained from the patient for publication of the block procedure and for use of patient data.

## Case presentation

Case 1

An anal fistulectomy was planned for 52-year-old man, American Society of Anesthesiologists (ASA) Classification II (weight = 98 kg, height = 177.8 cm) with no coexisting diseases. The patient had no unusual features in his medical history, and his blood tests, electrocardiograms, and a chest X-ray were evaluated as normal. He did not receive any medications. The risks and benefits of general anesthesia, spinal anesthesia, and sacral ESPB for surgical anesthesia were discussed with the patient. The patient adamantly refused general and spinal anesthesia, so sacral ESPB was decided for this patient. 

Case 2

A 46-year-old man ASA II Class (weight = 80 kg, height = 185 cm) and was scheduled for perianal fistulectomy. His medical history was significant for a 20 pack-year of cigarette smoking and ulcerative colitis. The patient was receiving mesalamine 500 mg every eight hours. Blood tests were normal, electrocardiograms showed sinus rhythm, and imaging (echocardiogram, chest X-ray, and computed tomography) appeared normal. The patient preferred sacral ESPB over general anesthesia since he had experienced severe postoperative nausea and vomiting during a previous surgery.

After standard monitoring in the operating room, the patients were placed in a prone position for the block procedure. In addition to oxygen (flow rate 2-3L/min), a midazolam bolus (IV 2 mg) was administered for sedation and a remifentanil infusion was started. The remifentanil infusion (0.05-0.1 μg/kg/min) was titrated to enable communication with patients (Ramsay Sedation Scale 2) throughout the surgery.

Description of the block technique

**Figure 1:**
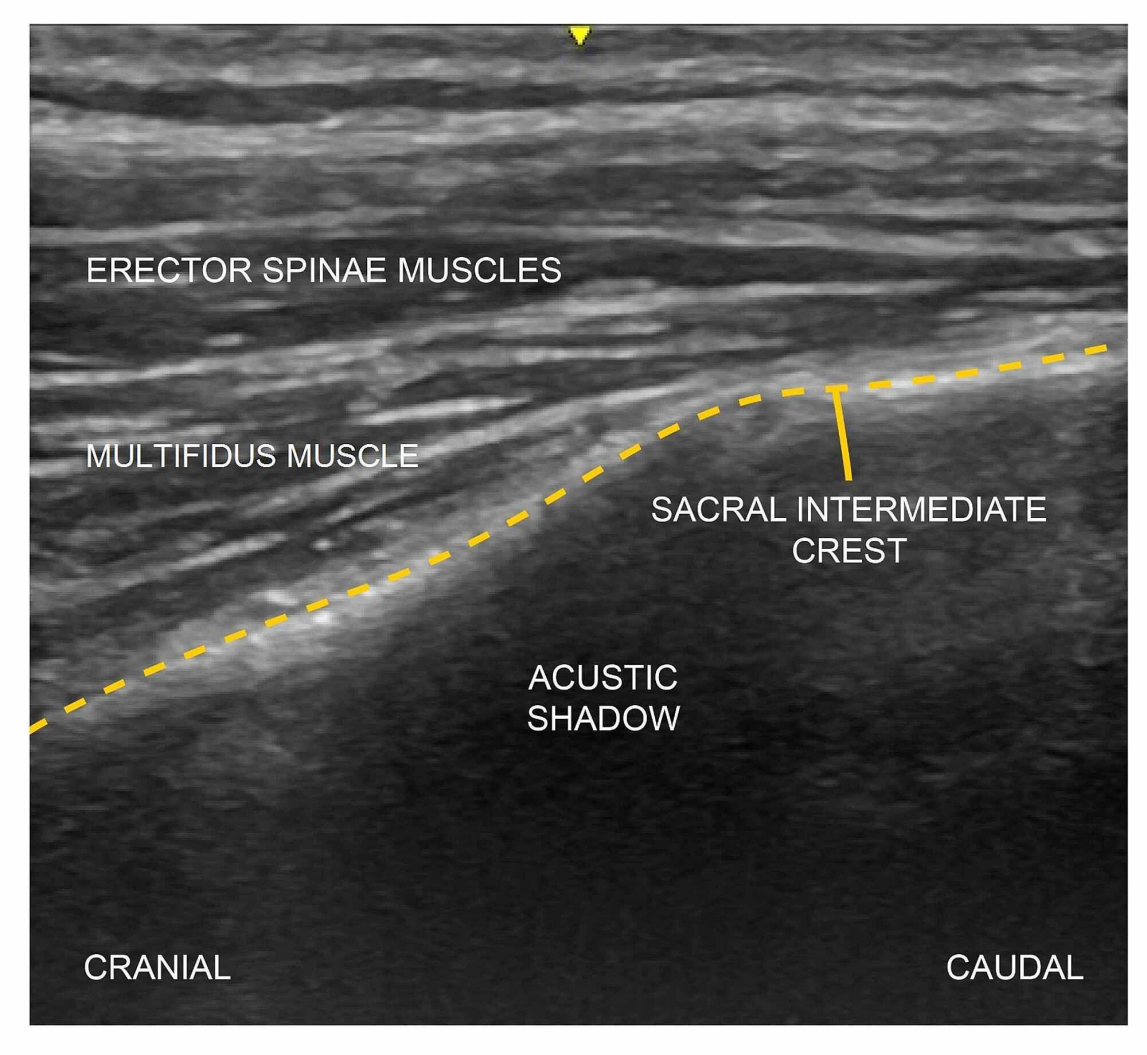
The sonoanatomy relevant for the ultrasound-guided sacral erector spinae plane block (ESPB). Dashed lines represent the sacral intermediate crest.

All patients were placed in the prone position. Aseptic conditions were provided for the block, and a linear ultrasound (US) probe (Logiq V1, GE, 8-13 MHz, China) was placed on the L5 vertebra spinous process in the sagittal plane to determine the beginning part of the sacrum. The probe was then moved caudally and the sacral medial crest was visualized. Here again, the probe was moved in the lateral direction until the sacral intermediate crest was visible (Figure 1). After obtaining the optimum image at the S2 level with the US, the block needle (100 mm, 21G short bevel; Stimuplex®, B. Braun, Germany) was advanced in the cranio-caudal direction as a plane, and bone contact was achieved. Prior to injection of the local anesthetic (LA) mixture (10 mL 0.5% bupivacaine + 10 ml 1% lidocaine), the needle was aspirated to exclude inadvertent intravascular placement of the needle tip. 10 mL was injected into the area between the multifidus muscle and the intermediate sacral crest. The probe was advanced slightly further in a caudal direction, the needle was redirected, and the remaining 10 mL LA mixture was injected at the S4 level. The same block procedure was repeated on the other side. During the block procedure, the carino-caudal propagation of the LA and the upward movement of the erector spinae muscle and multifidus muscle were observed in real time by the US. A sensorial blockade occurred in the S2-S5 dermatomes 30 min after the block procedure, and the surgical procedures were performed with the patients in the same position. Both patients remained hemodynamically stable throughout the surgery.

The patients were discharged at four hours postoperative without any complications (e.g., no muscle weakness in the lower extremities, and no urinary retention due to possible epidural extension). The patients, who were contacted later, indicated no need for any analgesic for 24 h postoperatively.

## Discussion

This case has shown us that bi-level, bilateral sacral ESPB can be an easy and effective alternative to surgical anesthesia in ambulatory anorectal surgeries. In fact, anorectal surgery is a procedure that requires deep anesthesia due to intense and multiple nerve innervation [7]. In cases where an adequate depth of anesthesia cannot be achieved, severe pain, uncontrolled movements, and laryngospasm (Brewer-Luckhardt reflex) can occur [8]. Local anesthesia (with or without intravenous sedation), regional anesthesia, and general anesthesia are frequently used for anesthesia in ambulatory anorectal surgeries [9]. General or spinal anesthesia could be another option in these cases, but postoperative pain, nausea-vomiting, respiratory depression with opioids, urinary retention, post-dural puncture headache, and their possible effects in delaying the patient's discharge should be kept in mind [10]. Ultimately, the decision was made to use sacral ESPB as an alternative because neither of our patients accepted these other techniques.

In the ESPB, LA injection is applied to the interfacial plane in the depth of the erector spinae muscle. The LA is spread in the cranio-caudal direction and provides multiple dermatomal blockades [11]. In anorectal surgery, S1, and especially S2-4, as well as S5 must be blocked to provide adequate visceral and somatic analgesia [12]. Therefore, to increase the LA spread in our cases, we preferred the technique of bilateral, bi-level (S2-S4) LA injection to the intermediate sacral crest area, rather than using a midline longitudinal approach [4,13].

Considering the mechanism of action, the sacral ESPB was first associated with blocking of the posterior divisions of the sacral nerves (S1-S3) [4]. However, an examination of the literature revealed that the sacral ESPB has been successfully used for postoperative analgesia in urological surgeries (gender reassignment, hypospadias repair) and anal surgeries (pilonidal sinus, anoplasty) [1,5,13,14]. In addition, our observation that the sacral ESPB can be used for surgical anesthesia as an alternative to general or spinal anesthesia in our cases leads us to think that the anterior divisions of the sacral nerves can be blocked, with possible epidural dissemination as the mechanism of action of the sacral ESPB.

Anatomically, when a sacral ESPB is performed, it is made on the plane between the LA multifidus muscle and the sacrum. Therefore, Hamilton later proposed that the block be named the “multifidus plane block,” based on the locations of the anatomical structures [15]. However, we prefer the designation “sacral ESPB” because many authors used this designation in the first descriptive and subsequent publications.

## Conclusions

In conclusion, we determined that the sacral ESPB can be safely used for surgical anesthesia under US guidance in our cases without causing any motor weakness or hemodynamic instability. This block can be considered in anorectal surgery as an alternative technique for spinal or general anesthesia. However, most of the literature information on its effectiveness is based on case reports. Therefore, randomized controlled trials are needed that investigate the analgesic efficacy/adverse effects. In addition, anatomical cadaver and imaging studies could also provide a better understanding of the mechanism of action.

## References

[1] Kukreja P, Deichmann P, Selph JP, Hebbard J, Kalagara H (2020). Sacral erector spinae plane block for gender reassignment surgery. Cureus.

[2] Topdagi Yilmaz EP, Oral Ahiskalioglu E, Ahiskalioglu A, Tulgar S, Aydin ME, Kumtepe Y (2020). A novel multimodal treatment method and pilot feasibility study for vaginismus: initial experience with the combination of sacral erector spinae plane block and progressive dilatation. Cureus.

[3] Ahiskalioglu A, Tulgar S, Celik M, Ozer Z, Alici HA, Aydin ME (2020). Lumbar erector spinae plane block as a main anesthetic method for hip surgery in high risk elderly patients: initial experience with a magnetic resonance imaging. Eurasian J Med.

[4] Tulgar S, Ahiskalioglu A, De Cassai A, Gurkan Y (2019). Efficacy of bilateral erector spinae plane block in the management of pain: current insights. J Pain Res.

[5] Tulgar S, Senturk O, Thomas DT, Deveci U, Ozer Z (2019). A new technique for sensory blockage of posterior branches of sacral nerves: ultrasound guided sacral erector spinae plane block. J Clin Anesth.

[6] Kılıcaslan A (2020). Bilevel-bilateral sacral erector spinae plane block for chronic pain management: a case report and short literature review. Selcuk Med J.

[7] (2019). Pelvis and perineum. Atlas of Human Anatomy.

[8] Suzuki M, Sasaki CT (1977). Effect of various sensory stimuli on reflex laryngeal adduction. Ann Otol Rhinol Laryngol.

[9] Diaz-Palacios GA, Eslava-Schmalbach JH (2011). Perirectal block for out-patient anorectal surgery: a new technique. Biomedica.

[10] Vinson-Bonnet B, Higuero T, Faucheron JL, Senejoux A, Pigot F, Siproudhis L (2015). Ambulatory haemorrhoidal surgery: systematic literature review and qualitative analysis. Int J Colorectal Dis.

[11] López MB, Cadórniga ÁG, González JML, Suárez ED, Carballo CL, Sobrino FP (2018). Erector spinae block: a narrative review. Cent Eur J Clin Res.

[12] Jorge JMN, Habr-Gama A (2007). Anatomy and embryology of the colon, rectum, and anus. The A.S.C.R.S. Textbook of Colon and Rectal Surgery.

[13] Aksu C, Gurkan Y (2020). Sacral erector spinae plane block with longitudinal midline approach: could it be the new era for pediatric postoperative analgesia?. J Clin Anesth.

[14] Oksuz G, Arslan M, Bilal B, Gisi G, Yavuz C (2020). Ultrasound guided sacral erector spinae block for postoperative analgesia in pediatric anoplasty surgeries. J Clin Anesth.

[15] Hamilton DL (2020). The erector spinae plane block: time for clarity over anatomical nomenclature. J Clin Anesth.

